# A 6-week, multicentre, randomised, double-blind, double-dummy, active-controlled, clinical safety study of lumiracoxib and rofecoxib in osteoarthritis patients

**DOI:** 10.1186/1471-2474-9-118

**Published:** 2008-09-08

**Authors:** Kirstin Stricker, Sue Yu, Gerhard Krammer

**Affiliations:** 1 Novartis Pharma AG Postfach, CH-4002, Basel, Switzerland; 2 Novartis Pharmaceuticals Corporation, One health Plaza East, Hanover, NJ, 07936-1080, USA

## Abstract

**Background:**

Lumiracoxib is a selective cyclooxygenase-2 inhibitor effective in the treatment of osteoarthritis (OA) with a superior gastrointestinal (GI) safety profile as compared to traditional non-steroidal anti-inflammatory drugs (NSAIDs, ibuprofen and naproxen). This safety study compared the GI tolerability, the blood pressure (BP) profile and the incidence of oedema with lumiracoxib and rofecoxib in the treatment of OA. Rofecoxib was withdrawn worldwide due to an associated increased risk of CV events and lumiracoxib has been withdrawn from Australia, Canada, Europe and a few other countries following reports of suspected adverse liver reactions.

**Methods:**

This randomised, double-blind study enrolled 309 patients (aged greater than or equal to 50 years) with primary OA across 51 centres in Europe. Patients were randomly allocated to receive either lumiracoxib 400 mg od (four times the recommended dose in OA) (*n *= 154) or rofecoxib 25 mg od (*n *= 155). The study was conducted for 6 weeks and assessments were performed at Weeks 3 and 6. The primary safety measures were the incidence of predefined GI adverse events (AEs) and peripheral oedema. The secondary safety measures included effect of treatment on the mean sitting systolic and diastolic blood pressure (msSBP and msDBP). Tolerability of lumiracoxib 400 mg was assessed by the incidence of AEs.

**Results:**

Lumiracoxib and rofecoxib displayed similar GI safety profiles with no statistically significant difference in predefined GI AEs between the two groups (43.5% *vs*. 37.4%, respectively). The incidence and severity of individual predefined GI AEs was comparable between the two groups. The incidence of peripheral oedema was low and identical in both the groups (*n *= 9, 5.8%). Only one patient in the lumiracoxib group and three patients in the rofecoxib group had a moderate or severe event. At Week 6 there was a significantly lower msSBP and msDBP in the lumiracoxib group compared to the rofecoxib group (*p *< 0.05). A similar percentage of patients in both groups showed an improvement in target joint pain and disease activity. The tolerability profile was similar in both the treatment groups.

**Conclusion:**

Lumiracoxib 400 mg od (four times the recommended dose in OA) provided a comparable GI safety profile to rofecoxib 25 mg od (therapeutic dose). However, lumiracoxib was associated with a significantly better BP profile as compared to rofecoxib.

**Trial registration number -:**

NCT00637949

## Background

Osteoarthritis (OA) is a common condition that affects 18% women and 10% men (aged > 60 years) worldwide [1]. Treatment for these patients is aimed at controlling pain, improving functional abilities and enhancing health-related quality-of-life [2].

Non-selective non-steroidal anti-inflammatory drugs (NSAIDs) such as naproxen and ibuprofen are widely used for pain relief in OA. However, upper gastrointestinal (GI) symptoms such as dyspepsia and more importantly ulcer complications occur in 15–60% of NSAID users and frequently necessitate co-therapy with H_2 _receptor antagonists or proton pump inhibitors [3-6]. In a prospective cohort study, it was observed that 81% of patients taking NSAIDs and having serious GI complications had no prior GI symptoms [7] and in a survey in the US among NSAID users, it was observed that nearly 75% of those who regularly used NSAIDs did not know about or were unconcerned about NSAID related GI complications [8]. GI adverse events (AEs) are the main factors limiting the use of NSAIDs and represent a significant health burden [6]. Renal impairment, vascular constriction and GI AEs are attributed to inhibition of cyclooxygenase-1 (COX-1), anti-inflammatory and analgesic effect is attributed to inhibition of COX-2. Hence, selective COX-2 inhibitors like celecoxib and rofecoxib provide a more favourable GI safety profile with similar efficacy as compared to non-selective NSAIDs in patients with OA [9,10]. Rofecoxib, however, was withdrawn worldwide on September 30, 2004 due to an increase in the cardiovascular (CV) risk [11]. Following this withdrawal, concerns have also been raised regarding CV safety of both selective COX-2 inhibitors and traditional NSAIDs. These concerns arose initially for selective COX-2 inhibitors following the worldwide withdrawal of rofecoxib. Meta-analyses have since reported an increased risk of CV events with both traditional NSAIDs and COX-2 inhibitors and both carry warnings to this effect in their prescribing information [12-14].

Lumiracoxib is a structurally distinct, selective COX-2 inhibitor for the management of OA and acute pain. Lumiracoxib is effective in treating acute pain conditions such as post-operative dental pain [15], acute gout [16], arthroplasty [17], sprains and strains [18] and in treating chronic pain associated with OA [19,20].

The 52-week Therapeutic Arthritis Research and Gastrointestinal Event Trial (TARGET) in 18 000 patients with OA investigated the GI, CV and overall safety profile of lumiracoxib 400 mg od (four times the recommended dose for OA) compared to two traditional NSAIDs, naproxen 500 mg bid and ibuprofen 800 mg tid [21,22]. The TARGET study showed that lumiracoxib was associated with a 79% decrease in upper GI complications compared to traditional NSAIDs (non-aspirin population) [21]. The GI benefit with lumiracoxib compared to traditional NSAIDs occurred within 8 days of treatment [23]. In TARGET lumiracoxib was also associated with an improved blood pressure (BP) profile as compared to the traditional NSAIDs, already after 4 weeks of treatment [24] and the effect was maintained until 52 weeks [22].

The present short-term safety study assessed the GI tolerability of a 6-week treatment with lumiracoxib 400 mg od (four times the recommended dose for OA) as compared to rofecoxib 25 mg od (therapeutic dose) in patients with OA. In addition, the study also assessed renal effects including the incidence of peripheral oedema and changes in BP in the two treatment groups.

## Methods

### Study design

This study was a 6-week, multicentre, randomised, double-blind, double-dummy, active-controlled, parallel-group, safety study of lumiracoxib 400 mg od (four times the recommended dose for OA) compared to rofecoxib 25 mg od. The study enrolled subjects with primary OA across 51 centres in Europe. This study was performed according to Good Clinical Practice guidelines. Ethics committee approval from all participating institutions was obtained in accordance with the Declaration of Helsinki and all patients gave their written informed consent before enrolment. The study had a 3–7 day wash-out period, 6-week treatment period and a follow-up by phone call 2 weeks after the end of study/early termination.

### Study population

Symptomatic patients (aged ≥50 years) with OA as defined by the American College of Rheumatology criteria were recruited. The criteria for inclusion were primary OA for at least 3 months in the hip, hand, knee or spine (cervical or lumbar) and pain in the target joint of at least moderate intensity (Likert scale). Patients also needed to be on NSAID or other analgesic therapy or expected to need NSAID treatment for at least 6 weeks.

The exclusion criteria were secondary OA and/or history/evidence of significant diseases in the affected joints, evidence of active ulceration or bleeding of the upper GI tract, upper GI tract malignancies, diseases of the intestinal tract and bleeding diathesis. Patients were excluded if they had clinically significant hepatic or renal disease, evidence of hepatic, renal or blood coagulation disorders or anaemia, hypertension, type I diabetes or other significant medical problems, used systemic steroids, intra-articular hyaluronic acid injections, H_2 _receptor antagonists, proton pump inhibitors, sucralfate or prostaglandin analogues in the past month. Pregnant or lactating women and women not on acceptable form of contraception were also excluded.

### Study medication and assessments

Patients were randomly allocated in the ratio of 1:1 to receive either lumiracoxib 400 mg od (four times the recommended dose for OA) or rofecoxib 25 mg od. Lumiracoxib (Prexige^® ^Novartis Pharma AG, Basel, Switzerland) was provided as 2 × 200 mg tablets with matching placebos and rofecoxib as 25 mg capsules with matching placebos. Patients were asked to take the medication once every morning at approximately the same time. Compliance with study drug was defined as patients taking ≥80% of the full daily dose. To control GI symptoms, patients were allowed a maximum of eight antacid tablets (calcium carbonate 680 mg/magnesium carbonate 80 mg) per day as rescue medication. Patients received the study medication for 6 weeks.

### Safety assessments

The key primary assessment was incidence of at least one of the predefined GI AEs: abdominal pain, constipation, diarrhoea, nausea, vomiting, dyspepsia and dysphagia. The other primary assessment was incidence of peripheral oedema: lower limb oedema, upper limb oedema, peripheral swelling and peripheral oedema. The secondary safety assessments were incidence of moderate and severe predefined GI AEs, incidence of each individual predefined GI AE, discontinuations from study because of any AE or GI AE and time to discontinuation, mean sitting systolic and diastolic blood pressure (msSBP and msDBP), and the number of tablets of antacid rescue medication taken. Study assessments were performed at baseline, Weeks 3 and 6.

Tolerability was evaluated by recording AEs during the entire study period. A follow-up phone call 2 weeks after the end of study was carried out to evaluate serious adverse events (SAEs) after study drug discontinuation. Investigators were requested to report all SAE's which occurred within 4 weeks after last dose of study drug intake. Vital signs including BP measurements and standard laboratory tests were performed at baseline, Weeks 3 and 6. ECG recordings were performed at baseline and Week 6.

### Efficacy assessment

Efficacy variables were overall pain intensity in the target joint and the global assessments of disease activity by patients and physicians on a 5-point Likert scale at Weeks 3 and 6. For overall pain intensity in the target joint, patients were classified as improved if endpoint assessment was "none" or improved by at least two grades from baseline on the Likert scale. For patient's and physician's global assessment of disease activity, patients were classified as improved if endpoint assessment was "very good" or improved by at least two grades from baseline.

### Statistical analysis

The categorical efficacy variables were analysed in the intent-to-treat (ITT) population defined as all randomised patients who received study medication. A multiple logistic model (PROC LOGISTIC in SAS), which considered treatment as main effect was used for the analysis. The treatment contrasts were tested at a two-sided 5% significance level and presented as odds ratios (ORs) together with their 95% confidence intervals (CIs). Missing data for efficacy variables were imputed using the last-observation-carried-forward (LOCF) method. The primary safety endpoints were analysed in the safety population defined as all patients randomised to treatment, who had been exposed to study medication. A multiple logistic model, which took into account country and treatment as the main effect was used for the analysis. The treatment contrasts were tested at a two-sided 5% level of significance and presented as ORs together with their 95% CIs. If the estimated incidence rates were less than 5%, or if the logistic regression model did not converge, Fisher's exact test was used for the comparisons. Analysis was repeated in the per-protocol (PP) population defined as a sub-population of the safety population for sensitivity reasons. Between-treatment comparisons for msSBP and msDBP were performed by means of analysis of covariance (ANCOVA). For the ANCOVA models, treatment and country were taken as fixed effects and the respective baseline values as covariate. Time to discontinuation from study due to any AE or GI AE was analysed using life-table methods. Patient compliance and other categorical safety endpoints were analysed using a multiple logistic model at a two-sided 5% significance level and presented as ORs together with their 95% CIs.

### Sample size and power considerations

The determination of the sample size was based on the key primary safety variable, the incidence of predefined GI AEs. A two-group continuity corrected chi-squared test with a two-sided 5% significance level had 80% power to detect a clinically relevant difference between the treatment groups, assuming 28% in the rofecoxib group and 14% in the lumiracoxib group when the sample size is 146 patients per treatment arm. Three hundred and four patients (152 each on lumiracoxib and rofecoxib) needed to be randomised to allow for a 4% dropout rate.

## Results

**Figure 1:**
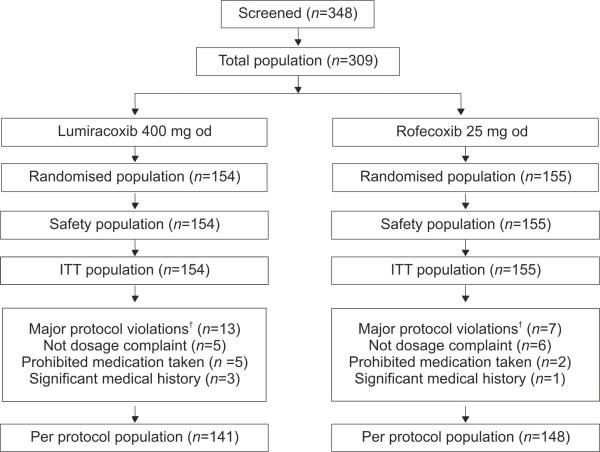
**Patient flow diagram**. †Patients with multiple occurrences of a major protocol violation (PV) were counted only once in that category of PV.

After an initial wash-out period of 3–7 days, a total of 309 patients were randomised to either lumiracoxib 400 mg od (*n *= 154) or rofecoxib 25 mg od (*n *= 155) (Figure 1). All randomised patients were included in the ITT and safety populations. At baseline, the treatment groups were comparable in terms of demographic and baseline characteristics (Table 1). Medical histories indicated that more patients on lumiracoxib had vascular disorders as compared to rofecoxib (54.5% *vs*. 46.5%, respectively). History of cardiac disorders at baseline was more frequent in lumiracoxib patients (16%) than rofecoxib patients (11%). In both the groups, a similar percentage (42%) of patients had previously undergone surgical and medical procedures. More than 90% of patients in both the treatment groups completed the study. Major protocol violations resulting in exclusion from the PP population occurred in 13 patients receiving lumiracoxib and seven patients receiving rofecoxib.

**Table 1:** Demographic and baseline characteristics (safety population)

	**Lumiracoxib 400 mg od** **(*n *= 154)**	**Rofecoxib 25 mg od** **(*n *= 155)**
Age (years)^†^	65.3 ± 8.49	65.5 ± 8.67
Women, *n *(%)	94 (61.0)	100 (64.5)
BMI (kg/m^2^)^†^	29.1 ± 5.21	28.3 ± 4.51
Race		
Caucasians, *n *(%)	154 (100.0)	153 (98.7)
Other	0 (0.0)	2 (1.2)
Disease duration (years)^†^	7.41 ± 7.058	8.37 ± 8.407
Physician's global assessment of disease activity *n *(%)		
Very good	1 (0.6)	2 (1.3)
Good	3 (1.9)	5 (3.2)
Fair	75 (48.7)	66 (42.6)
Poor	69 (44.8)	76 (49.0)
Very poor	6 (3.9)	6 (3.9)
Patient's global assessment of disease activity *n *(%)		
Very good	1 (0.6)	1 (0.6)
Good	9 (5.8)	6 (3.9)
Fair	51 (33.1)	57 (36.8)
Poor	78 (50.6)	77 (49.7)
Very poor	15 (9.7)	14 (9.0)
Pain intensity assessment *n *(%)		
Moderate	74 (48.1)	74 (47.7)
Severe	67 (43.5)	67 (43.2)
Extreme	13 (8.4)	14 (9.0)
Current smokers, *n *(%)	24 (15.6)	26 (16.8)

BMI = body mass index; Other = Black/African American and Asian or Pacific islander; SD = standard deviation.

^†^Mean ± SD

### Primary safety endpoints

**Figure 2:**
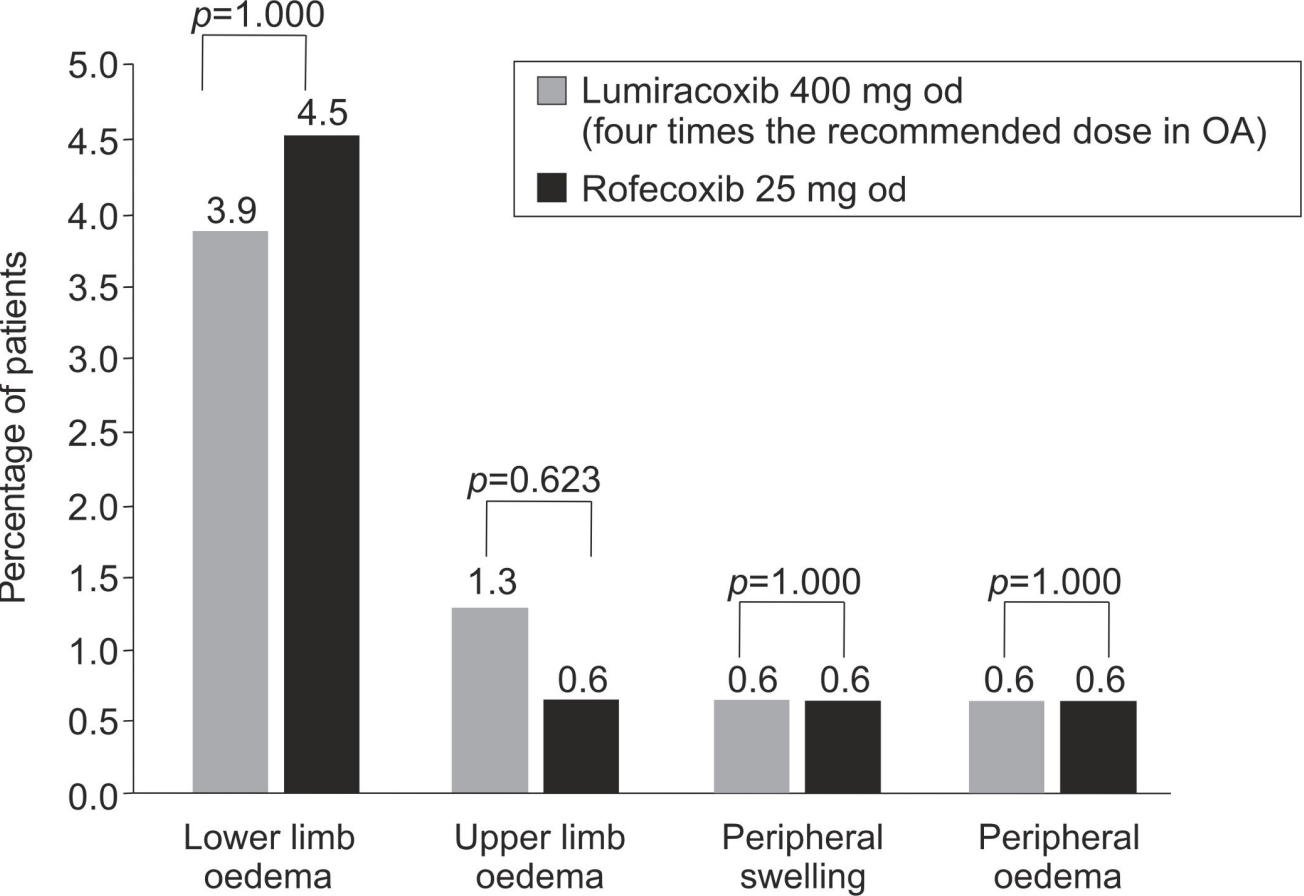
**Incidence of peripheral oedema in patients treated with lumiracoxib and rofecoxib (safety population)**. The incidence of peripheral oedema at Week 6. Pairwise comparisons tested at the two-sided 5% significance level. *p*-value computed using Fisher's exact test. OA, osteoarthritis.

There was no statistically significant difference in the overall incidence of key primary assessment variables (predefined GI AEs – abdominal pain, constipation, diarrhea, nausea, vomiting, dyspepsia and dysphagia) between the treatment groups (OR: 1.31; 95% CI: 0.82, 2.11, *p *= 0.258). Predefined GI AEs were reported in 43.5% (*n *= 67) of patients in lumiracoxib group and 37.4% (*n *= 58) of patients in rofecoxib group. Thus, overall both the study drugs displayed similar GI safety profiles. The incidence of the other primary assessment variable, peripheral oedema was low in both the treatment groups (*n *= 9, 5.8%) (Figure 2).

### Secondary safety endpoints

There was no statistically significant difference between lumiracoxib and rofecoxib for the incidence of individual predefined GI AEs. Minor differences between lumiracoxib and rofecoxib in the incidence rates for diarrhoea (11.0% *vs*. 5.2%), dyspepsia (26.6% *vs*. 20.6%) and constipation (2.6% *vs*. 0.6%) were observed, but were not statistically significant. When the incidence rates of these predefined GI AEs were analysed based on their severity it was observed that moderate or severe predefined GI AEs associated with lumiracoxib and rofecoxib were comparable with the exception of dyspepsia that occurred more often in the lumiracoxib group (11.0% lumiracoxib *vs*. 4.5% rofecoxib, *p *= 0.035, Fisher's exact test [Table 2]).

**Table 2:** Incidence of moderate or severe predefined GI AEs (safety population)

**Predefined GI AEs**	**Lumiracoxib 400 mg od** **(*n *= 154)** ***n *(%)**	**Rofecoxib 25 mg od** **(*n *= 155)** ***n *(%)**
Abdominal pain	6 (3.9)	6 (3.9)
Constipation	1 (0.6)	1 (0.6)
Diarrhoea	4 (2.6)	2 (1.3)
Dyspepsia*	17 (11.0)	7 (4.5)
Dysphagia	1 (0.6)	0 (0.0)
Nausea	3 (1.9)	0 (0.0)
Vomiting	1 (0.6)	1 (0.6)

GI = gastrointestinal; AEs = adverse events

*p = 0.032

**Figure 3:**
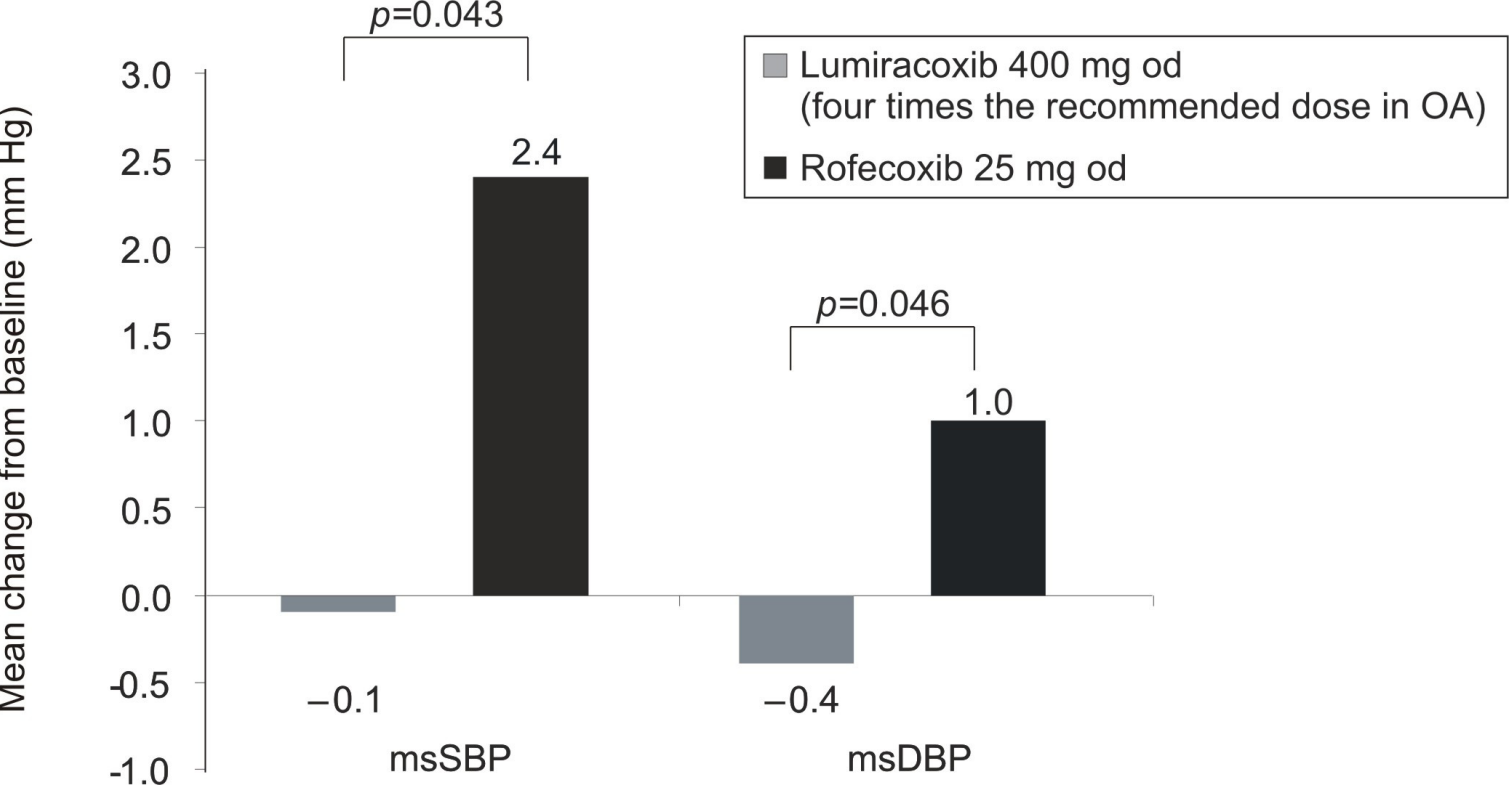
**Lumiracoxib shows better blood pressure profile as compared to rofecoxib (safety population)**. msSBP – Mean sitting systolic blood pressure. msDBP – Mean sitting diastolic blood pressure. *p*-value computed from ANCOVA on mean blood pressure at Day 42 with centre, treatment, and baseline blood pressure value. Mean change from baseline at Week 6. OA, osteoarthritis.

The rate of moderate-to-severe peripheral oedema was low in both treatment groups. Only one patient in the lumiracoxib group (0.6%) *versus *three patients (1.9%) in the rofecoxib group had a moderate-to-severe event. This numerical difference was not statistically significant. After 6 weeks of treatment, a significantly lower msSBP and msDBP was observed with lumiracoxib as compared to rofecoxib (least square estimated difference: -3.13 mmHg, 95% CI: – 6.17, -0.10, *p *= 0.043 for msSBP and -1.73 mmHg, 95% CI: -3.43, – 0.03, for msDBP, *p *= 0.046 [Figure 3]). The mean number of antacid tablets taken was the same in both treatment groups (0.2 tablets/day).

The most frequently reported AEs (by preferred term) during this study are listed in Table 3. The incidence of AEs was comparable between lumiracoxib and rofecoxib. The most commonly reported AEs by primary system organ class were GI disorders, infections and infestations, and musculoskeletal and connective tissue disorders, which were similar in incidence in both treatment groups. Study-drug related AEs as suspected by the investigator were reported in 40.9% of patients in the lumiracoxib group and 37.4% of patients in the rofecoxib group. As expected in a study focussing on GI safety, AEs were most commonly reported in the GI system. Three rofecoxib-treated patients experienced AEs that led to temporary interruption of study medication (Table 4).

**Table 3:** Incidence of most frequent AEs (≥2% for either group) by preferred term (safety population)

	**Lumiracoxib 400 mg od** **(*n *= 154)** ***n *(%)**	**Rofecoxib 25 mg od** **(*n *= 155)** ***n *(%)**
Dyspepsia	41 (26.6)	33 (21.3)
Abdominal pain NOS	15 (9.7)	10 (6.5)
Diarrhoea NOS	15 (9.7)	7 (4.5)
Nausea	8 (5.2)	8 (5.2)
Abdominal pain upper	4 (2.6)	7 (4.5)
Constipation	4 (2.6)	1 (0.6)
Oedema lower limb	6 (3.9)	7 (4.5)
Fatigue	5 (3.2)	4 (2.6)
Nasopharyngitis	9 (5.8)	9 (5.8)
Influenza	6 (3.9)	6 (3.9)

AEs = adverse events; NOS = not otherwise specified

**Table 4:** Incidence of deaths and SAEs (Safety population)

	**Lumiracoxib 400 mg od** **(*n *= 154)**	**Rofecoxib 25 mg od** **(*n *= 155)**
Patients with serious AEs		
Death *n *(%)	0 (0.0)	0 (0.0)
Non-fatal SAEs *n *(%)	0 (0.0)	1 (0.6)
Patients with other significant AEs		
Pre-specified AEs (GI events or oedema) *n *(%)	73 (47.4)	64 (41.3)
AEs leading to dose adjustment/interruption *n *(%)	0 (0.0)	3 (1.9)
Discontinuation due to		
Any AEs including SAEs *n *(%)	8 (5.2)	7 (4.5)
SAEs *n *(%)	0 (0.0)	0 (0.0)
AEs (non-serious) *n *(%)	8 (5.2)	7 (4.5)

AEs = adverse events; SAEs = serious adverse events; GI = gastrointestinal

Discontinuations due to GI AEs occurred in 4.5% and 2.6% of the patients treated with lumiracoxib and rofecoxib, respectively (*p *= 0.359). The mean time to discontinuation for patients treated with lumiracoxib as compared to rofecoxib for any AE (23.3 days *vs*. 21.7 days, respectively) and for GI AEs (23.4 days *vs*. 25.3 days, respectively) was comparable. A similar proportion of patients discontinued from the study due to any AE in both groups (5.2% of lumiracoxib and 4.5% of rofecoxib patients).

No drug-related SAEs or deaths were reported during the course of the study. One SAE (vaginal haemorrhage) was reported in the rofecoxib group.

Serum chemistry and haematology parameters were in the normal range at baseline for the majority of patients in both the treatment groups and remained so at the end of study. No elevations in alanine amino transferase/aspartate amino transferase > 3 × ULN were observed during the study.

### Efficacy endpoint

An improvement in target joint pain or disease activity was reported in 30–40% of patients in the lumiracoxib and rofecoxib groups after 6 weeks of treatment (Table 5). The differences between the treatment groups were not statistically significant for any efficacy parameter.

**Table 5:** Efficacy results in patients treated with lumiracoxib and rofecoxib (ITT population)

	**Week 3**		**Week 6**	
**Efficacy measures**	**Lumiracoxib 400 mg od** **(*n *= 154)**	**Rofecoxib 25 mg od** **(*n *= 155)**	**Lumiracoxib 400 mg od** **(*n *= 154)**	**Rofecoxib 25 mg od** **(*n *= 155)**

Patient's pain intensity				
Improved *n *(%)	53 (34.4)	50 (32.3)	49 (31.8)	63 (40.6)
Non-improved *n *(%)	101 (65.6)	105 (67.7)	105 (68.2)	92 (59.4)
Patient's global assessment of disease activity				
Improved *n *(%)	49 (31.8)	53 (34.2)	57 (37.0)	65 (41.9)
Non-improved *n *(%)	105 (68.2)	102 (65.8)	97 (63.0)	90 (58.1)
Physician's global assessment of disease activity				
Improved *n *(%)	44 (28.6)	46 (29.7)	51 (33.1)	56 (36.1)
Non-improved *n *(%)	110 (71.4)	109 (70.3)	103 (66.9)	99 (63.9)

## Discussion

In this study, both lumiracoxib and rofecoxib showed similar efficacy in treating pain associated with OA.

The GI safety profile of lumiracoxib 400 mg od (four times the recommended dose for OA) was comparable to rofecoxib 25 mg od over 6 weeks of treatment. The incidence of individual predefined GI AEs and their severity was also comparable between the treatment groups.

Lumiracoxib is indicated at a dose of 100 mg once daily for chronic use in OA, and at doses of 200 mg or 400 mg once daily for short-term use in acute pain indications. While liver toxicity is a known rare but serious side effect of all COX-2 inhibitors and traditional NSAIDs [25], there have been some specific concerns from health authorities regarding the hepatic safety profile of lumiracoxib. Lumiracoxib was withdrawn in Australia in August 2007 following reports of severe liver events occurring predominantly at doses higher than the recommended dose of 100 mg od, when taken chronically. The US FDA issued a non-approvable letter in September 2007, citing concerns over the hepatic profile of lumiracoxib. This was followed by withdrawals in Canada, Europe and a few other countries. Assessment of the benefit to risk profile of the drug is currently ongoing by a number of health authorities.

Liver toxicity is a known rare but serious side effect of all COX-2 inhibitors and traditional NSAIDs and it is not clear the risk is higher with lumiracoxib than other NSAIDs.

In this 6-weeks study no elevations in liver enzymes were observed with lumiracoxib. This is in agreement with the results from TARGET where the incidence of ALT/AST elevations > 3 × ULN were low with lumiracoxib, comparable to ibuprofen and naproxen and no "Hy's cases" (ALT/AST > 3 × ULN and total bilirubin > 3 mg/dL), which are more predictive for severe liver outcome, were observed during the first 49 days of treatment [26].

Traditional NSAIDs and selective COX-2 inhibitors like rofecoxib and etoricoxib have been shown to increase BP in clinical studies [27,28] and in the recent Multinational Etoricoxib and Diclofenac Arthritis Long-Term (MEDAL) study, discontinuations due to hypertension were observed more frequently with etoricoxib compared with diclofenac [29]. In this study, after 6 weeks of treatment, a statistically significantly better BP profile was observed with lumiracoxib as compared to rofecoxib, with an estimated difference of more than 3 mmHg systolic blood pressure (SBP) in favour of lumiracoxib. Although this difference was small, reports suggest that increases in SBP of 1–5 mmHg have been associated with 7100–35 700 additional ischemic heart disease and stroke events in OA patients over a 1-year period in the USA [30]. These findings are consistent with previous findings where a 2 mmHg decrease in SBP reduced the risk of death due to ischemic heart disease and stroke by approximately 7% and 10%, respectively, in middle age [31]. Hence, maintaining BP control can provide substantial benefits in OA patients [32].

These results are in agreement with the findings of the 12-month TARGET outcome study with lumiracoxib, where lumiracoxib had an improved BP profile compared with ibuprofen or naproxen [22,24]. The improved BP profile with lumiracoxib as compared to ibuprofen was also observed in hypertensive OA patients [33]. In addition, results from a meta-analysis involving 9 611 patients on lumiracoxib (100–400 mg od) revealed that lumiracoxib provided a BP profile (both systolic and diastolic) comparable to placebo [34].

Moreover, in TARGET, the incidence of oedema was low and lumiracoxib was not associated with any increase in the incidence of oedema, compared with ibuprofen or naproxen [35], while in the VIGOR study, the incidence of oedema was higher in the rofecoxib group as compared to the naproxen group [36]. The incidence of peripheral oedema was low and similar in both the groups in this study. A numerical difference for moderate and severe peripheral oedemas was also observed in favour of lumiracoxib, although it did not reach statistical significance.

The incidence of AEs and discontinuations due to AEs were comparable between the treatment groups. The most common AEs suspected by the investigator to be study-drug related were GI AEs, as expected in a study on GI safety.

## Conclusion

Lumiracoxib 400 mg od (four times the recommended dose in OA) demonstrated comparable GI safety profile to rofecoxib 25 mg od (therapeutic dose) in patients with OA. However, lumiracoxib was associated with a significantly better BP profile as compared to rofecoxib.

## Abbreviations

AEs: adverse events; ANCOVA: analysis of covariance; BP: blood pressure; CIs: confidence intervals; COX: cyclooxygenase; CV: cardiovascular; GI: gastrointestinal; ITT: intent-to-treat; LOCF: last-observation-carried-forward; MEDAL: Multinational Etoricoxib and Diclofenac Arthritis Long-Term; msDBP: mean sitting diastolic blood pressure; msSBP: mean sitting systolic blood pressure; NSAIDs: non-steroidal anti-inflammatory drugs; OA: osteoarthritis; Ors: odds ratios; PP: per-protocol; SAEs: serious adverse events; SBP: systolic blood pressure; TARGET: Therapeutic Arthritis Research and Gastrointestinal Event Trial.

## Appendix 1: List of Investigators

**Austria**: Dr. Winfried Graninger, Universitaetsklinik fuer Innere Medizin III, Klin. Abteilung Rheumatologie, Waehringer Guertel 18–20, A-109 Vienna; Dr. Peter Peichl, Kaiser-Franz-Josef-Spital, 2. Medizinische Abteilung mit Rheumatologie und Osteologie der Stadt Wien, Kundratstrasse 3, A-1100 Wien; Dr. Attila Dunky, Wilhelminenspital der Stadt Wien, 5. Mediz. Abteilung m. Rheumatologie, Stoffwechsel, Rehabilitation Montleartstrasse 37, A-11650 Vienna; Dr. Josef Hermann, Medizinische Universitaets Klinik, Universitaet Graz Auenbruggerplatz 15, 8036 Graz

**Belgium**: Prof. P. Geusens, Biomedisch Onderzoeksinstituut – DWI, Limburgs Universitair Centrum, Universitaire Campus – Building C, 3590 Diepenbeek; Prof. Jean-Pierre Devogelaer, Cliniques Universitaire St. Luc, Service de Rhumatologie, Avenue Hippocrate 10, 1200 Bruxelles

**France**: Dr. C. Copere, Private Practice, Roanne; Dr. A. Duplain, Private Practice, Roanne; Dr. D. Estienne, Private Practice, Roanne; Dr. M. Fleury, Private Practice, Roanne; Dr. P.L. Jacquier, Private Practice, Roanne; Dr. J. Richard, Private Practice, Roanne; Dr. J.M. Aupy, Private Practice, Roanne; Dr. S. Benayoune, Private Practice, Roanne; Dr. J.-M. Blot, Private Practice, Roanne; Dr. D. Brechoire, Private Practice, Roanne; Dr. M. Gacioch, Private Practice, Roanne; Dr. G. Etchegary, Private Practice, Niort; Dr. C. Tilly, Private Practice, Niort; Dr. P. Amlard, Private Practice, Niort; Dr. M. Anthony, Private Practice, Niort; Dr. M. Baert, Private Practice, Niort; Dr. J. Marty, Private Practice, Murs Erigne; Dr. J-F. Pascal, Private Practice, Murs Erigne; Dr. D. Tirouflet, Private Practice, Murs Erigne

**Netherlands**: Dr. G.J.M. van Doesburg, Private Practice, Lichtenvoorde; Dr. W.A. de Backer, Private Practice, Rijswijk; Dr. C.P. Buiks, Private Practice, Ewijk; Dr. H.F.C.M. Van Mierlo, General Practice Van Mierlo Rembrandt, van Rijn Singel 37-c, 2371 RB Roelofarendsveen; Dr. A. Veerman, Private Practice, Huizen; Dr. M. Passage, Private Practice, Kerkrade

**Switzerland**: Dr. med. Hans-Ulrich Rentsch, Rheumatologie, Poststrasse 25, 9000 St Gallen; Dr. R Theiler, Kantonsspital Aarau, Buchserstrasse/Haus 1, 5001 Aarau; Dr. med. Hans Schwarz, Rheumatologie Bethesda-Spital, Gellertstrasse 144, 4020 Basel; Dr. med. Michel Pellaton, 2, ruelle du Peyrou, 2000 Neuchâtel; Dr. Heinz Fahrer, Lindenhofspital/Rheumatologische Klinik, Salihaus Bremgartenstrasse 117, 3012 Bern; Dr. Ottmar Gorschewsky, Klinik Permanence Bern West, Orthopädie Bümplizstrasse 83, 3018 Bern; Dr. Thomas Lehmann, Inselspital/Rheumatologische Klinik Eingang-EG-29/Eingang 14a Freiburgstrasse, 3010 Bern; Dr. Paul Hasler, Felix Platter Spital, Rheumatologie Burgfelderstr. 101, 4012 Basel; Dr. med. Pierre-Alain Buchard, Rhumatologie FMH Clinique romande de réadaptation, Avenue Grand Champsec 90, 1951 Sion; Dr. med. Jean Dudler, Hôspital Nestlé, CHUV Rhumatologie FMH Avenue Pierre-Decker 5, 1005 Lausanne; Dr. Pierre-André Guerne, HCUG Rhumatolgoie, FMH Avenue Beau Séjour 25, 1211 Genève 14; Dr. med. Daniel Uebelhart, Universitätsspital Zürich Gloriastrasse 25, 8091 Zürich; Dr. Urs Moser, Rheumatologie Mühlegasse 3, 4410 Liestal; Dr. med. Michel Braun, Rhumatolgoie FMH Rue Gustave-Amweg 21, 2900 Porrentury

**United Kingdom**: Dr Alun George, The Staploe Medical Centre, The Staploe Medical Centre Brewhouse Lane Soham, CB7 5JD Cambridge; Dr Duncan Burwood, Bedgrove Surgery, Bedgrove Surgery Brentwood Way, HP21 7TL Aylesbury; Dr Andrew Cowie, The Porch Beechfield Road, Corsham, SN13 9 Wiltshire; Dr Robert Matthews, The Spa Surgery, The Spa Surgery 6 Spa Road, SN12 7NS Melksham; Dr Anthony Wright, Hathaway Surgery, Hathaway Surgery 32 New Road, SN15 1 Chippenham; Dr Kevin Gruffydd-Jones, Box Surgery, Box Surgery London Road, SN13 8NA Box, Corsham, Wiltshire

## Competing interests

SY is an employee of Novartis Pharmaceuticals Corporation, East Hanover, NJ. KS and GK are employee of Novartis Pharma AG, Basel, Switzerland (the manufacturer of lumiracoxib). All authors own stocks of the company.

## Authors' contributions

KS and GK participated in analysis and interpretation of the data. Godehard Hoexter performed the original statistical analysis which was then used by SY to conduct the statistical review of this manuscript. All authors contributed to drafting the manuscript. All authors read and approved the final manuscript.

## Pre-publication history

The pre-publication history for this paper can be accessed here:


